# The brain networks indices associated with the human perception of comfort in static force exertion tasks

**DOI:** 10.3389/fnrgo.2025.1542393

**Published:** 2025-05-30

**Authors:** Lina Ismail, Waldemar Karwowski

**Affiliations:** ^1^Arab Academy for Science, Technology and Maritime Transports, Department of Industrial and Management Engineering, Alexandria, Egypt; ^2^Computational Neuroergonomics Laboratory, Department of Industrial Engineering and Management Systems, University of Central Florida, Orlando, FL, United States

**Keywords:** brain network, coherence, EEG, network indices, perception of physical comfort, static force exertion, arm flexion

## Abstract

**Introduction:**

The perception of physical comfort is one of the important workplace design parameters. Most comfort perception studies have mainly relied on subjective assessments and biomechanical techniques, with limited exploration of neural brain activity.

**Methodology:**

The current study investigates this research gap by integrating the rating of perceiving physical comfort (RPPC) with brain network indices in an arm flexion task across different force levels. The applied arm forces, EEG-based neural responses, and the RPPC were measured, and the corresponding network theory indices were calculated. The following correlations were evaluated: (a) RPPC and applied forces, (b) network theory indices and applied forces, and (c) RPPC and network theory indices.

**Results and discussion:**

Results for (a) revealed a significant negative correlation between RPPC and the applied force for the arm flexion task. This shows that as the exerted force difficulty increases to an extremely hard level, the perception of physical comfort decreases till it reaches no comfort level. Results for (b) showed a positive correlation between the applied forces and global efficiency for the alpha network coherence during an extremely hard task. In contrast, a negative correlation was found between applied forces and path length for beta coherence during a light task. Findings from (b) suggest that the brain is more efficient in transmitting information related to cognitive functioning during a highly demanding force exertion task than a light task. Results from (c) showed a negative correlation between RPPC and global efficiency for alpha coherence during an extremely hard force exertion task. Moreover, a positive correlation was observed between RPPC and local efficiency for beta coherence during a somewhat hard task. Findings from (c) also indicate that perceiving a low-comfort physical task might increase task alertness, with the corresponding neural network exhibiting a high level of internal brain organization.

**Conclusions:**

The study results contribute valuable knowledge toward understanding the neural responses underlying the perception of physical comfort levels.

## 1 Introduction

Comfort is a complex concept that can be defined from different perspectives, including physical, psychological, sociological, and technological (Dumur et al., 2004). Physical comfort relates to the absence of pain or discomfort. Psychologically, comfort involves the feeling of wellbeing, safety, satisfaction, or relaxation perceived by humans in a working environment. Sociologically comfort encompasses a sense of support and acceptance of that individual experience. Technological comfort involves the role of technology in enhancing comfort. Designing a comfortable physical environment in the workplace reduces health risk issues resulting mainly from work-related musculoskeletal disorders (WMSDs). WMSDs are one of the most frequent disorders in occupational health, leading to long-term sick leave. Ensuring a comfortable environment in the workplace is a very complex task since it depends on human's psychological and physiological states and the surrounding environmental conditions (Slater, 1985). Despite the numerous previous ergonomics studies addressing comfort, the perception of comfort during physical activity continues to be insufficiently understood. Perceptions have a measurable effect on the sense of physical comfort (Rahman et al., 2023). Measuring the perception of comfort during a physical task continues to be insufficient for decoding the whole perception (Alessandro et al., 2014) because it is affected by subjective judgment (Richard, 1980; Hernandez et al., 2002). Previous studies have claimed that objective measures have several advantages compared to subjective measurements for assessing comfort (Lee et al., 1993). Although a large body of knowledge about objective measures has been gathered over the last decades, the neurophysiological bases remain poorly understood, leading to the ignorance of some useful brain information (Shortz et al., 2012). Therefore, the current study significantly contributes to the existing body of knowledge on the neurophysiological basis of physical comfort perception by investigating the relationship between physical comfort and brain activity during various force levels.

Network Science provides advances in understanding complex phenomena of any system (Watts and Strogatz, 1998; Amaral and Ottino, 2004). In network science, a brain network is modeled as a graph G (N, E), with N denoting the number of nodes that are connected through edges E in graph G. Nodes represent individual neurons or brain regions, while edges represent nodes interactions (Newman, 2003). Edges convey the brain connectome classified into structural, functional, and effective brain connectivity (Sporns et al., 2005). Functional connectivity analyzes the statistical dependence among brain regions on a fast time scale (Sporns, 2013), especially after advances in neuroimaging techniques, mainly electrophysiological (EEG) techniques.

Different functional connectivity analysis methods and their interpretational pitfalls are reviewed in Bastos and Schoffelen (2016). The Coherence method is a promising approach for estimating the functional connectivity patterns and interactions of brain data (Andrew and Pfurtscheller, 1999; Canteroa et al., 1999; Nolte et al., 2004; Sauseng et al., 2005; Comani et al., 2013; Bowyer, 2016; Storti et al., 2016). Previous studies have demonstrated its effectiveness in various areas, such as evaluating physiological abnormalities (Adler et al., 2003; Wang et al., 2014), quantifying executive processes (Sauseng et al., 2005), assessing pain levels (Modares-Haghighi et al., 2021), detecting mental fatigue (Qi et al., 2020), studying motor learning (Dal Maso et al., 2018) and evaluating performance in physical tasks (Di Fronso et al., 2018; Tamburro et al., 2020; Visser et al., 2024). For instance, Di Fronso et al. (2018) found higher EEG coherence values at rest than cycling across all electrodes pairs. This indicates that focusing attention stimulates various parts of the brain region and improves the participant's performance in physical activity. However, a sustained mental task increases EEG coherence but does not improve the performance efficiency (Chen et al., 2014). Another study showed a higher alpha EEG coherence in both bilateral parietal-frontal and parietal-central regions in successful compared to unsuccessful golf put activity (Babiloni et al., 2008).

**Figure 1:**
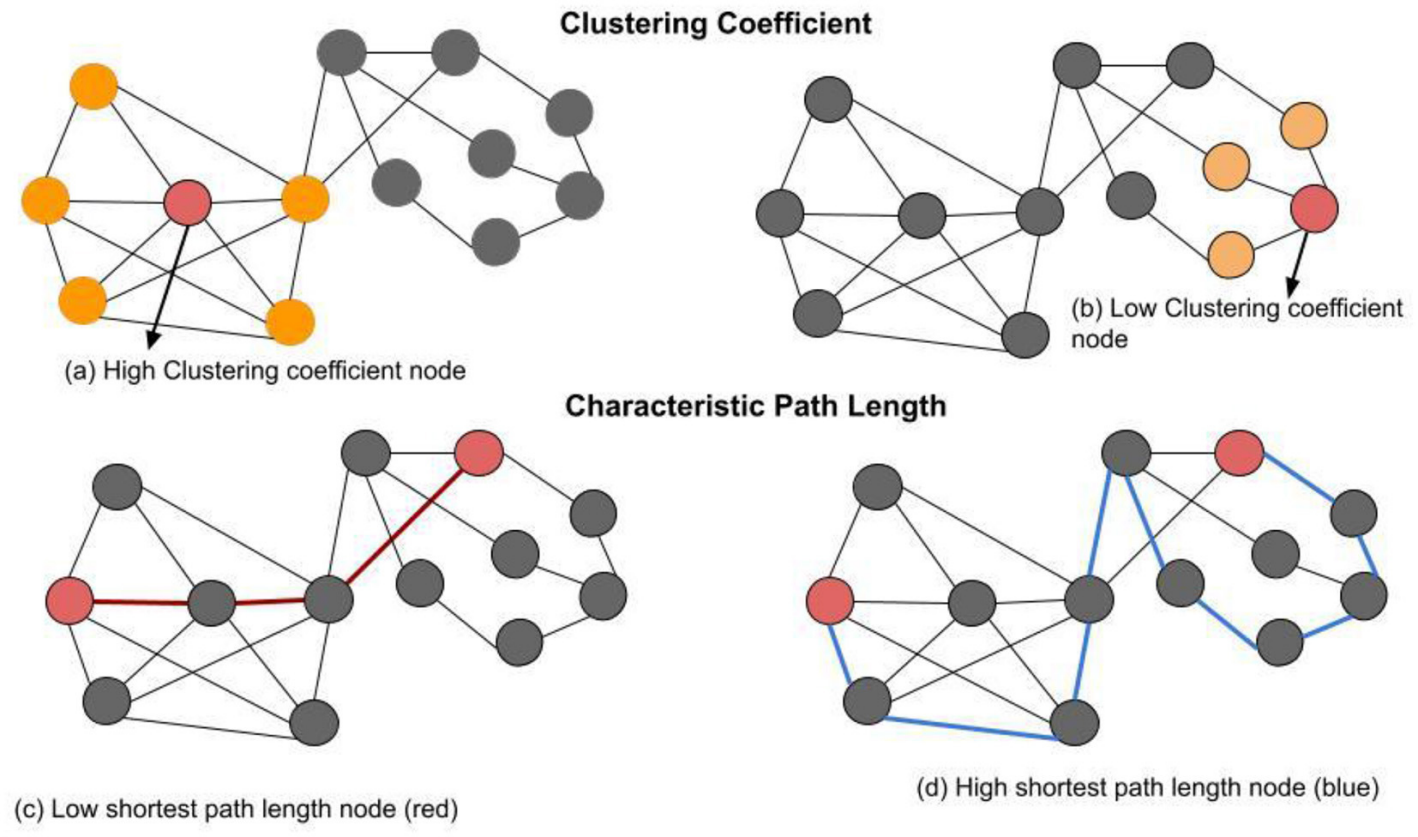
Basic network metrics: A network with 13 nodes and 22 edges. **(a)** The node labeled with “high clustering coefficient node” (red) has in total of five neighbors (orange) that are connected by seven existing edges between neighbors only. Thus, the clustering coefficient of the labeled node is (0.71). **(b)** The node labeled with “low clustering coefficient node” (red) has in total of three neighbor's nodes (oranges) that are not connected to each other. Thus, the clustering coefficient value of this node is 0, because there are no existing edges among its three neighbors (oranges). **(c)** Two red nodes are connected to each other by three edges (red lines), this is an indication of a low shortest path length between these specific two nodes. **(d)** Two red nodes are connected to each other by nine edges (blue lines), this is an indication of a high shortest path length between these specific two nodes.

The application of Graph theory is promising for analyzing the brain network through mathematic models represented as graphs (Stam and Reijneveld, 2007; Bullmore and Sporns, 2009). Various network measures quantifies meaningful information regarding the brain network topological properties (Bastos and Schoffelen, 2016; Sporns and Betzel, 2016; Vecchio et al., 2017; Sporns, 2018; Farahani et al., 2019; Ismail and Karwowski, 2020). Four network indices are commonly used, namely average clustering coefficient, characteristic path length, global network efficiency, and local network efficiency. The clustering coefficient represents the extent to which a node's neighbors are also connected to each other, forming a clique. A high clustering coefficient node has neighbors that are highly connected. In Figure 1a, the node labeled with “high clustering coefficient node” (red) has a total of five neighbor's nodes (orange) that are connected by seven existing edges between neighbors only. A low clustering coefficient node has neighbors that are lightly connected to each other or no connection at all. In Figure 1b, the node labeled with “low clustering coefficient node” (red) has a total of three neighbor nodes that are not connected. The characteristic path length is the global average distance between all pairs of network nodes. Figure 1c, the two red nodes are connected to each other by three edges in red. This is an indication of a low shortest path length between these specific two nodes. Figure 1d, shows the same two nodes connected to each other by nine edges in blue. This is an indication of a high shortest path length between these specific two nodes. Global network efficiency measures the network efficiency of transmitting information. Local efficiency measures the efficiency of information integrated between the neighbor networks in the local subgraph.

The study investigates the relationship between human perception of comfort and brain activity regarding brain network indices in static force flexion tasks. This study provides a novel application of network indices in comfort research contributing to the existing body of knowledge on the neurophysiological basis of comfort perception, which had not been explicitly explored in prior research.

This work is an extension of a previous study we presented in Ismail et al. (2022). In this regard, the current study aims to answer the following research questions (RQ):

- RQ1: Whether a correlation exists between subjective scores using RPPC and applied forces?
- RQ2: Whether a correlation exists between the applied forces and brain network indices?
- RQ3: Whether a correlation exists between subjective scores using RPPC and brain network indices?

To the best of our knowledge, this is the first study exploring the neural correlation of the perception of physical comfort during physical activity utilizing global and local network indices for two frequency bands, alpha, and beta, in healthy female participants.

The rest of the paper is organized as follows: Methodology is presented in Section 2; The data collection includes anthropometric data, applied force data, subjective comfort scoring and brain network indices in Section 3. The results for three correlation analysis are explained in Section 4. The discussions and limitations are in Section 5. Finally, Section 6 demonstrates the study limitations and considerations. Finally, Section 7 concludes the study.

## 2 Methodology

### 2.1 Location of data collection and participants

The data were collected in a computational neuroergonomics lab at the University of Central Florida, USA. The Institutional Review Board approved the study. Twelve female participants (mean age 28 ± 6 years) who met the inclusion criteria were recruited. The study focused on female subjects only to minimize variability related to gender based neurophysiological and biomechanical differences that could confound the interpretation of brain connectivity and comfort perception under physical activity.

This design choice was particularly important for a preliminary investigation seeking to explore novel neurophysiological correlations of physical comfort.

Exclusion criteria included pregnancy, neurological or psychological disorders, history of cardiovascular problems, chronic physical disorders, no exercise within 48 h before EEG recording, and no consumption of coffee or alcohol for 24 h before EEG recording. All the participants provided informed consent and demonstrated an adequate understanding of the study procedure.

### 2.2 Experiment design and protocol

The study was designed to experimentally measure the participants' (a) EEG signals, (b) applied forces, and (c) comfort levels during an arm flexion task for female subjects at five predefined levels of exertion selected based on Borg scale (Borg, 1982). Participates were provided a standardized instructions before conducting the experiment and were given brief description on how to interpret and use the RPPC scale to precisely use it and reduce response bias. Participants were firstly requiring applying the maximum voluntary contraction (MVC) for 3 s for three trials, with a 30 s rest period between each trial. Then, participants were asked to apply force by pulling the chain upward for 3 s by using their flexed arms without any body movement. This was repeated three times with a 30-s rest period between them. Random sequences of force levels were used to avoid potential learning biases. This protocol has been previously used by Chaffin et al. (1978). The experiment was designed on three trials in which each participant performed three repetitions of arm flexion force then ratings collected comfort level after each trial. The final RPPC for each force level was averaged across trials for reducing the influence of outlier responses and increasing statistical reliability. The arm flexion task was performed with the Jackson Strength Evaluation System (Jackson, 1994), and applied force was collected using a TORBAL FC5k force measurement device calibrated in Newton (N). Then, participants assessed their comfort levels using an 11-point unidimensional comfort scale as follows: (0 = No comfort; 1= very low comfortable; 3 = fair comfortable; 5 = moderate comfort; 6 = more than moderate comfort; 8 = high comfortable; 10 = very high comfortable). This subjective physical comfort scale has been developed specifically to measure physical comfort in manual handling tasks and has been validated in previous studies (Karwowski et al., 1992, 1999; Genaidy et al., 1998; Kee and Karwowski, 2001a,b; Yeung et al., 2002; Rahman et al., 2023; Diniz et al., 2024).

### 2.3 EEG recording and preprocessing

64 Ag-AgCl scalp electrodes were set on the scalp according to the international 10–20 system, and the linked earlobe was used as a reference. During the data acquisition, participants were instructed to avoid unnecessary body movements or eye blinks. The recorded data were acquired using Cognionics acquisition software (Cognionics, Inc, 2024). A proposed EEG data preprocessing pipeline proposed by Ismail et al. (2022). Impedance was maintained below 10 Ω, and signals were sampled at 500 Hz with a bandpass filter of 0.1–100 Hz. EEG time series data were pre-processed using EEGLAB (version 14.1.2b; Delorme and Makeig, 2004), an open-source toolbox that runs on MATLAB R2019b software (MathWorks, Natick, MA). EEG cross spectra were extracted based on Fast Fourier Transform using Hanning windows with 10% onset. The cross spectra were averaged across the 50% overlapping windows, considering two frequency bands: alpha (8–13 Hz) and beta (13–30 Hz) for each participant.

All experiments were conducting in a temperature-controlled laboratory to minimize environmental variability and brain artifacts.

## 3 Data collection

### 3.1 Anthropometric data

The mean value for all participants anthropometric data were calculated as follows: age 27.4 years, body weight 60.2 kg, shoulder height 135.85 cm, hip height 98.04 cm, Knee height 51.65, arm height 106.26 cm, knuckle height 73.98 cm, and body height 163 cm.

### 3.2 Force flexion data

After collecting the force flexion measures from all participants, the mean and the standard deviations were calculated through the 3 s period of exerted force in each condition as percentages of each participant's MVC as follows: extremely hard (67.35, 35.25), hard (41.83,18.9), somewhat hard (34.58, 16.7), light (13.61, 6.76), and extremely light (8.04, 5.32). This ensures that exertion levels were relative to individual physical capabilities rather than absolute force values which helped to normalize the data across participants.

### 3.3 Rate of perceived comfort

Subjective comfort scores were collected from all participants by ranking their rate of perceived physical comfort (RPPC). The mean and standard deviations of RPPC at each window trial for three trials at each applied force level were as follows: extremely light (8.23, 2.30), light (7.750, 2.11), somewhat hard (5.729, 1.68), hard (5.375, 1.63), and extremely hard (4.583, 1.48).

### 3.4 Coherence calculations and brain network indices

**Figure 2:**
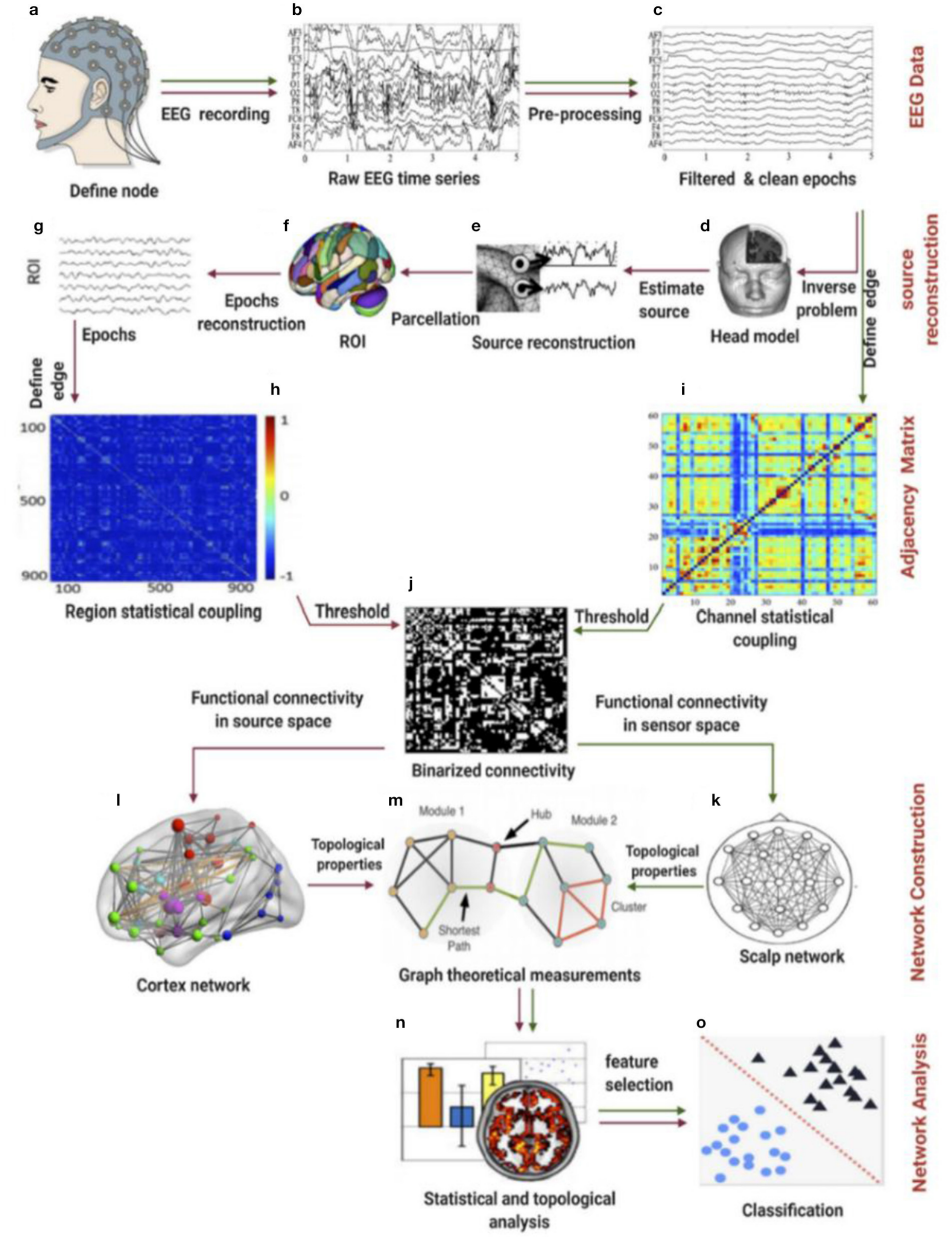
Schematic illustration of the pipeline for the construction of a functional brain network based on EEG data using graph theory. The green line defines the first approach, termed the “sensor signal” or “individual channels” method, while the red line defines the second approach, denoted as “EEG source connectivity.” **(a)** Place the cap containing electrodes on the scalp. **(b)** Record the EEG time series. **(c)** Preprocess the data by cleaning, filtering, removing artifacts, and epoching. **(d)** Solve the inverse problem by first estimating or imaging the head model (method 2). **(e)** Reconstruct the electrical potential time source (method 2). **(f)** Parcel the source reconstructed epochs into the ROI (method 2). **(g)** Define the ROI for the epochs. **(h)** Develop the connectivity matrix for the selected ROI. **(i)** Develop the connectivity matrix for the selected EEG channels (method 1). **(j)** Apply the threshold value(s) to binarize the connectivity matrix (methods 1 & 2). **(k)** Construct the scalp functional brain network between EEG electrodes. **(l)** Construct the cortex functional brain network within the ROI. **(m)** Apply the network topological properties to calculate graph theory measurements. **(n)** Apply statistical analysis methods. **(o)** Classify different states, if needed.

A proposed pipeline by Ismail and Karwowski (2020) has been used to calculate brain functional connectivity and network indices (Figure 2). The coherence method was selected to obtain the functional Connectivity for alpha and beta networks using an exact low-resolution brain electromagnetic tomography, an inverse solution with exact zero localization error (Pascual-Marqui, 2007). Four network indices were selected to characterize each network's topological properties: clustering coefficient, characteristic path length, global efficiency, and local efficiency. The Clustering coefficient (CC) is used to describe the degree to which a node in the graph tends to cluster together; the Local efficiency (El) measures how well a node is connected to its neighbors in a network; the characteristic path length (PL) is the average minimum distance traveled between two nodes for all possible pairs in a network; the global efficiency (Eg) is the inverse of the average distances between all pairs of nodes (Watts and Strogatz, 1998; Newman, 2003). Brain network indices were calculated using the Brain Connectivity Toolbox (http://www.brain-connectivity-toolbox.net) (Rubinov and Sporns, 2010). Network coefficient values, including clustering coefficient, path length, global efficiency, and local efficiency for the alpha network at each force level, including extremely hard, hard, somewhat hard, light, and extremely light, are summarized in Table 1. Network coefficient values, including clustering coefficient, path length, global efficiency, and local efficiency for the beta network at each force level, including extremely hard, hard, somewhat hard, light, and extremely light, are summarized in Table 2.

**Table 1:** Network coefficient values for different force levels for alpha coherence network.

**Applied Force**	**Clustering coefficient (CC)**	**Path length (PL)**	**Global efficiency (Eg)**	**Local efficiency (El)**
Extremely hard	0.2516	1.436	0.1715	0.278
Hard	0.251	1.446	0.1694	0.2765
Somewhat hard	0.2508	1.448	0.1694	0.276
Light	0.251	1.448	0.1694	0.2765
Extremely light	0.251	1.446	0.1695	0.2765

**Table 2:** Network coefficient values for different force levels for beta coherence network.

**Applied Force**	**Clustering coefficient (CC)**	**Path length (PL)**	**Global efficiency (Eg)**	**Local efficiency (El)**
Extremely hard	0.2445	0.89205	0.2522	0.3238
Hard	0.244	0.89195	0.2524	0.3235
Somewhat hard	0.2441	0.89202	0.2523	0.32355
Light	0.2441	0.89203	0.2523	0.32345
Extremely light	0.2439	0.8919	0.25235	0.3234

## 4 Results and analysis

Sstatistical analyses were conducted using Minitab for spearman correlation and Python for Locally Estimated Scatterplot Smoothing. Given the ordinal nature of the comfort ratings (RPPC) and the relatively small sample size (*n* = 12), a nonparametric method to examine the relationships between subjective comfort rating, applied force, and brain network indices. Spearman's rank correlation and Locally Estimated Scatterplot Smoothing (LOESS) analyses were conducted. Spearman's correlation was used to quantify monotonic associations, while LOESS was applied to visually assess potential nonlinear trends. These nonparametric statistical tests are suitable for small sample sizes to mitigate assumptions about normality and enhance the robustness of the findings.

### 4.1 Correlation between the RPPC and applied forces

**Figure 3:**
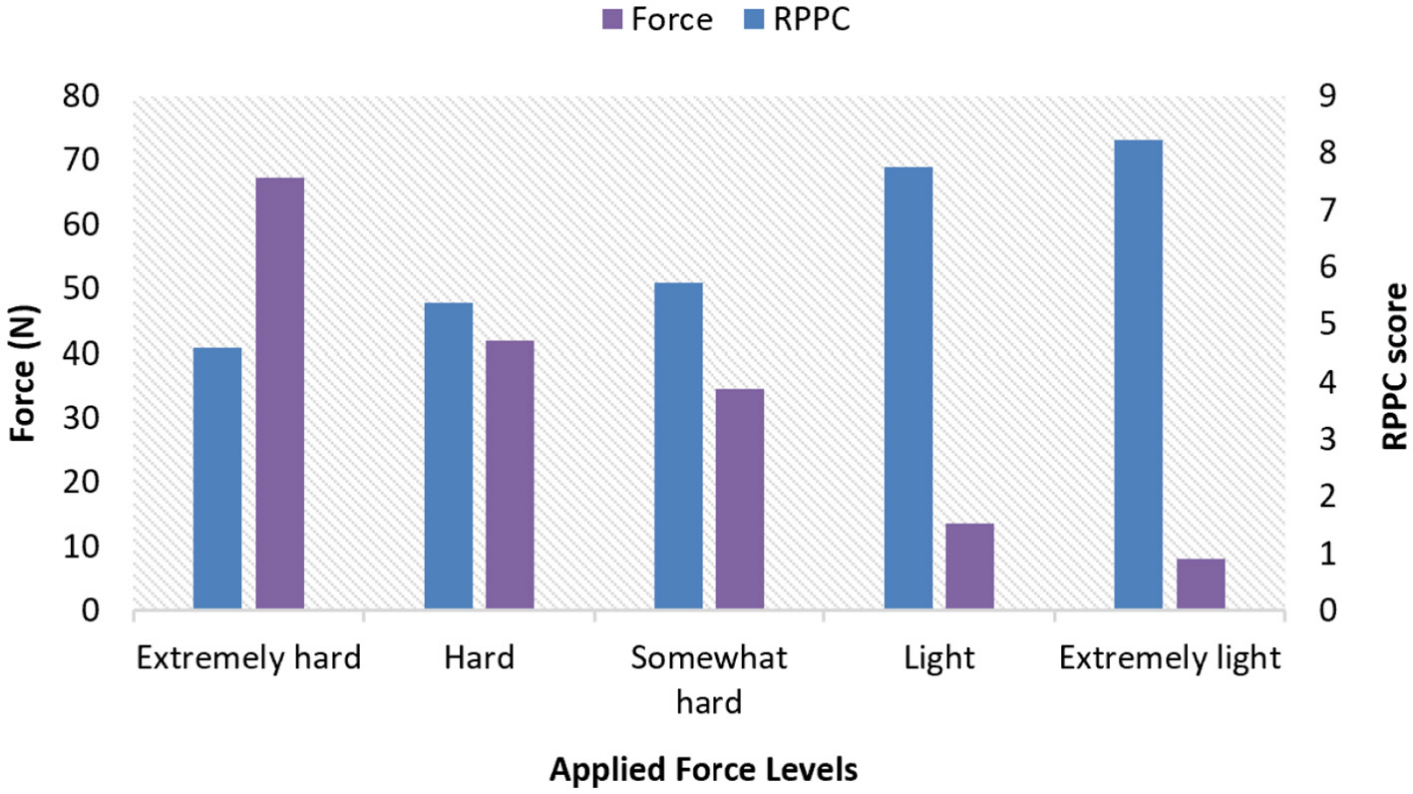
Applied arm forces and rate of perceived physical comfort (RPPC) scores bar plot at different applied force levels across all subjects.

**Figure 4:**
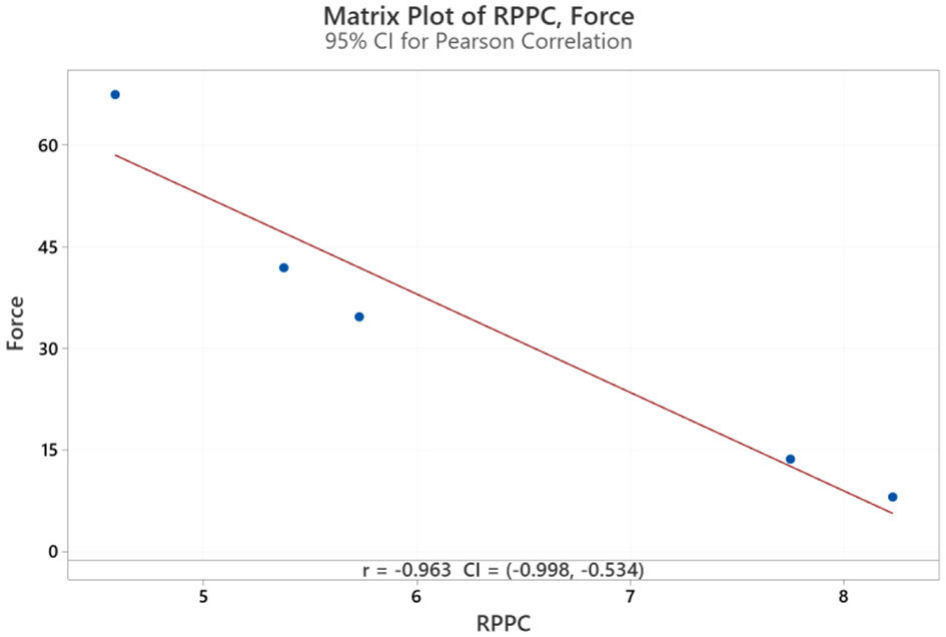
Correlation of rate of perceived physical comfort (RPPC) and force applied in Newton.

**Figure 5:**
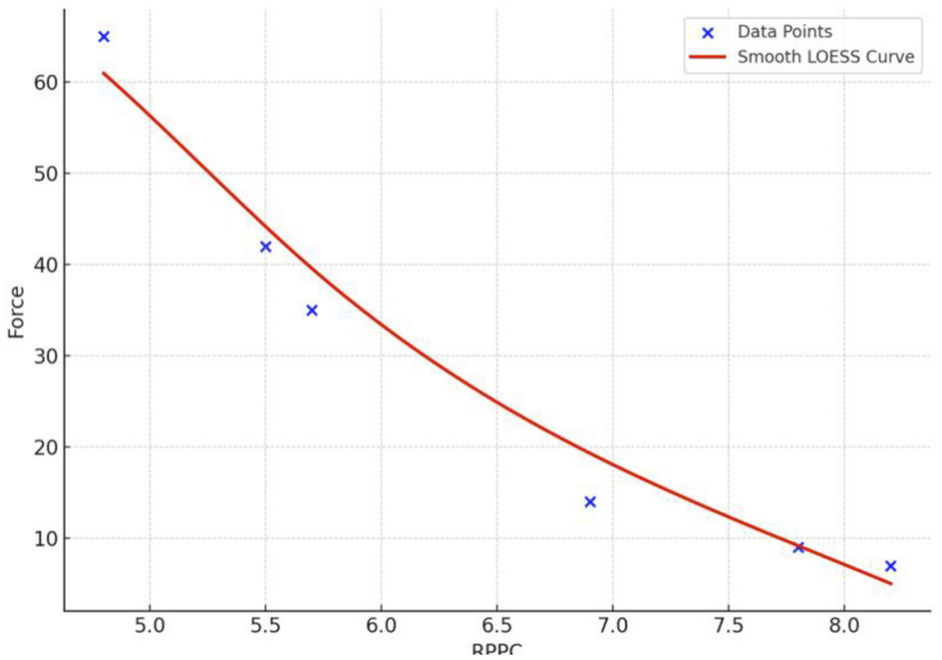
The LOESS curve of rate of perceived physical comfort (RPPC) and force applied in Newton.

The results for all arm applied forces and related RPPC scores at five applied force levels across all subjects (Figure 3). Spearman correlation coefficients were calculated to determine whether there is a correlation between RPPC and the applied forces (addressing RQ1). The spearman correlation analysis revealed a significant negative correlation between the RPPC and applied force (*r* = −0.963; *p* < 0.01) (Figure 4). The LOESS curve supports that comfort and force have an inverse relationship but not necessarily linear (Figure 5).

### 4.2 Correlation between applied force and network indices

**Figure 6:**
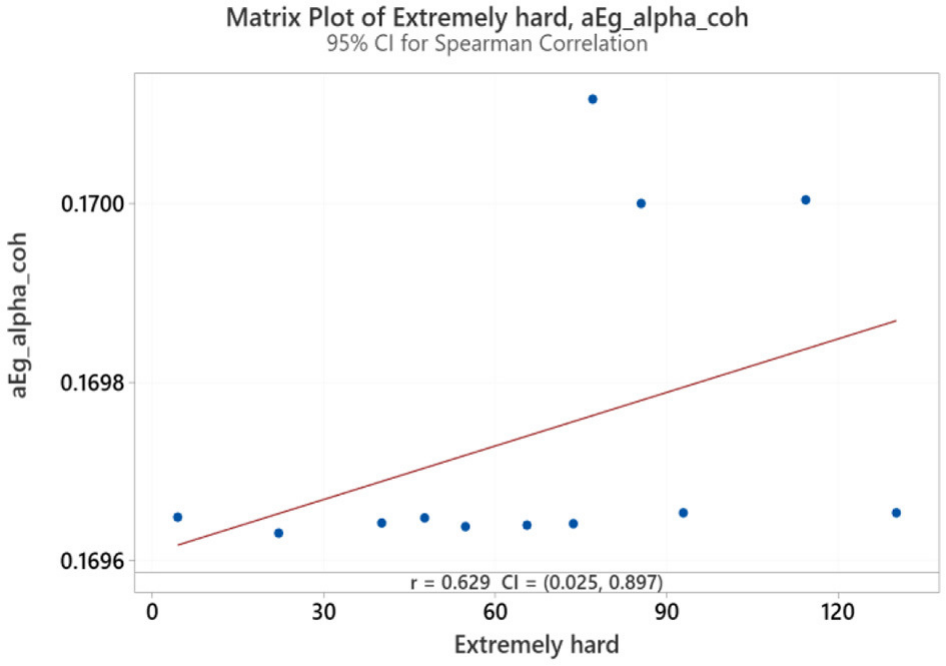
Correlation between extremely hard applied force and global efficiency for alpha coherence network (aEg_alpha_coh).

**Figure 7:**
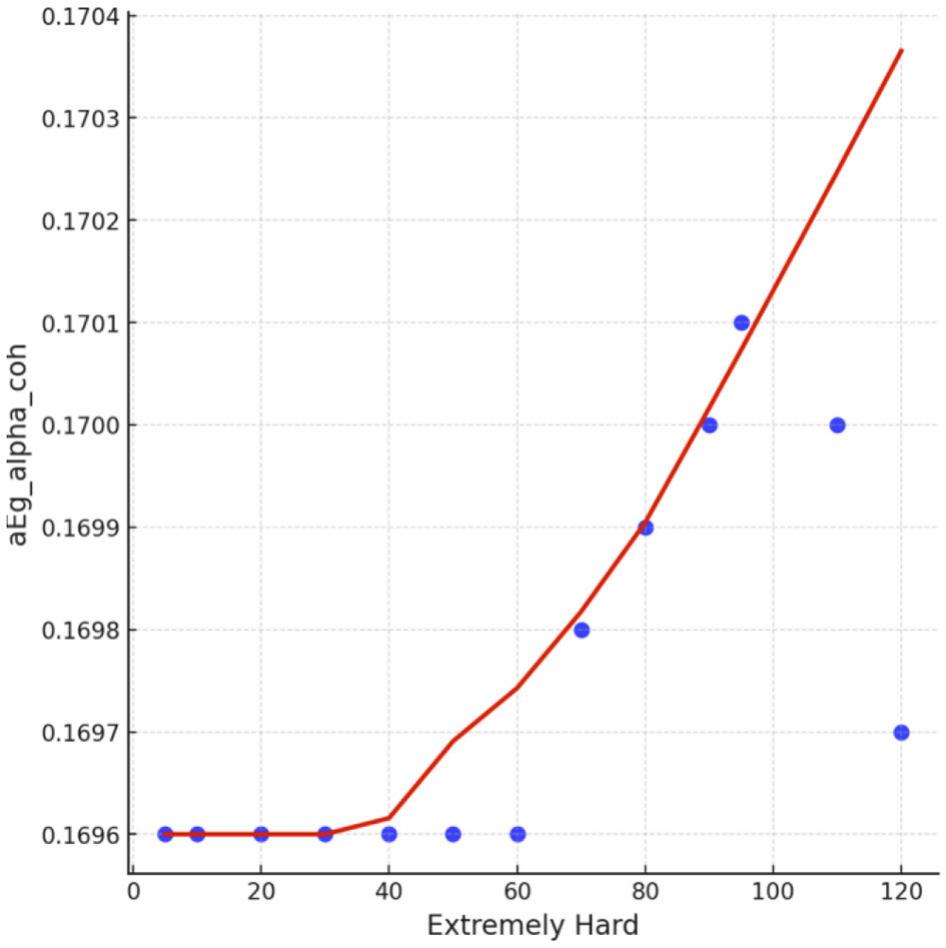
The LOESS curve of extremely hard applied force and global efficiency for alpha coherence network (aEg_alpha_coh).

**Figure 8:**
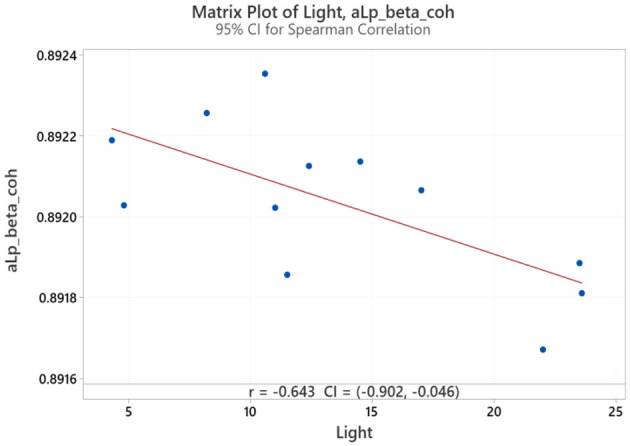
Correlation between light applied force and path length for beta coherence network (aLp_beta_coh).

**Figure 9:**
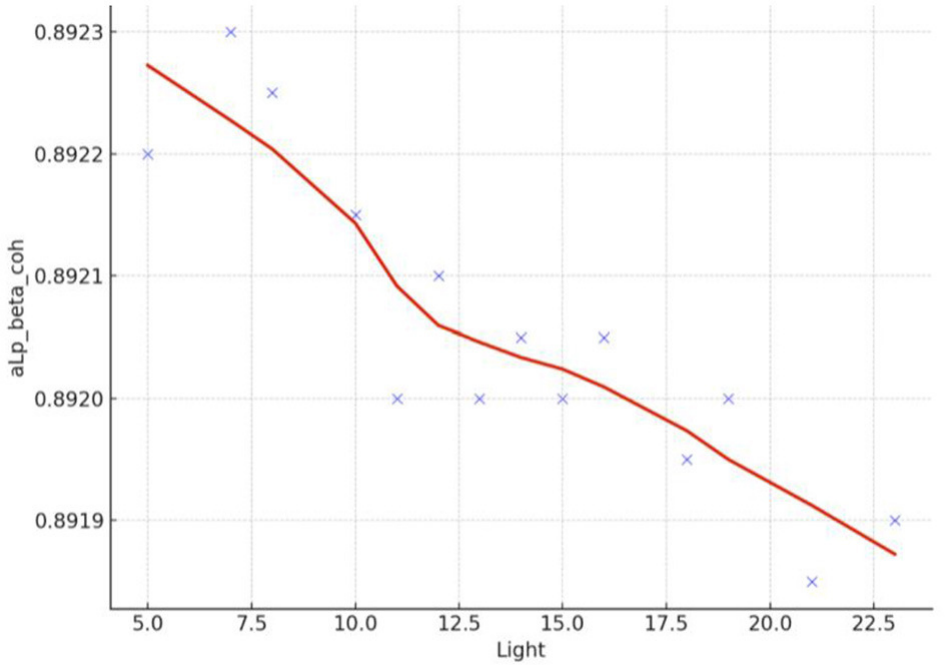
LOESS curve of light applied force and path length for beta coherence network (aLp_beta_coh).

Spearman correlation coefficients were calculated to investigate the possible relationship between applied forces (N) and network Indices (addressing RQ2). Significant results were found only for global efficiency in the alpha coherence network and the path length for the beta coherence network for two applied forces. The extremely hard level of applied force positively correlated with global efficiency in the alpha coherence network (*r* = 0.629, *p* = 0.028, Figure 6) but did not correlate with any other network indices. Figure 7 shows that the alpha coherence remains stable at lower applied force levels and shows a nonlinear increase in middle force levels. However, at the extremely hard level a slight decrease occurs, suggesting a diminishing return in network efficiency under extreme physical stress. At the light force level, there was a negative correlation with path length in beta coherence network (*r* = −0.643, *p* = 0.024, Figure 8). No significant correlations were found between network indices and the other applied forces, including somewhat hard, hard, and extremely light levels. Figure 9 demonstrates that LOESS curve is not perfectly straight for the light force level and path length in beta coherence network.

### 4.3 Correlation between RPPC and network indices

**Figure 10:**
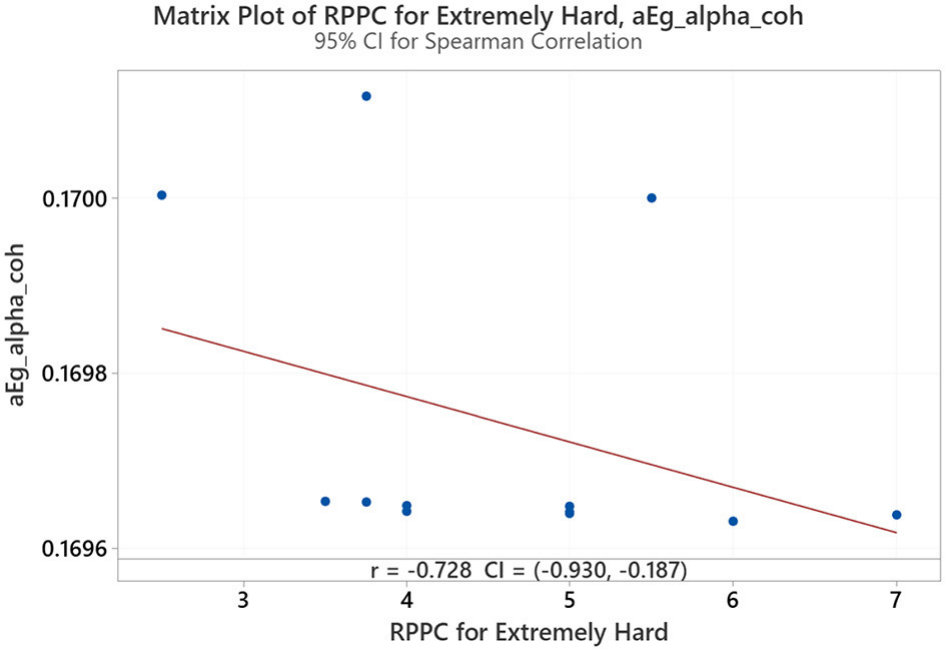
The correlation between the RPPC at extremely hard applied force and global efficiency for alpha coherence network (aEg_alpha_coh).

**Figure 11:**
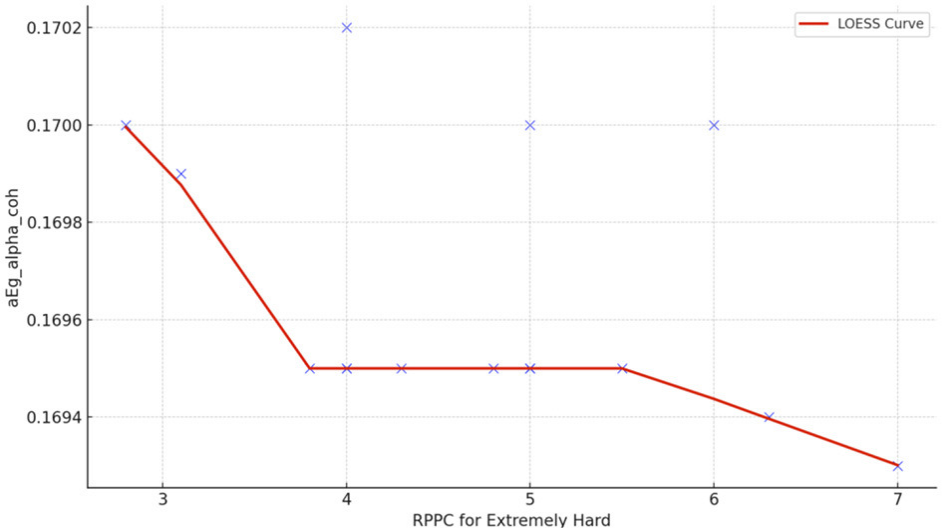
The LOESS curve of RPPC at extremely hard applied force and global efficiency for alpha coherence network (aEg_alpha_coh).

Spearman correlation coefficients were calculated to investigate a possible relationship between the RPPC and network indices (addressing RQ3). Meaningful results were only found extremely hard and somewhat hard. At the extremely hard applied force level, negative correlations were observed between comfort scores and global efficiency for alpha coherence (Figure 10). Results from LOESS curve shows a negative trend where low RPPC is associated with lower alpha network coherence, possibly due to increased task demand related to applied force (Figure 11).

**Figure 12:**
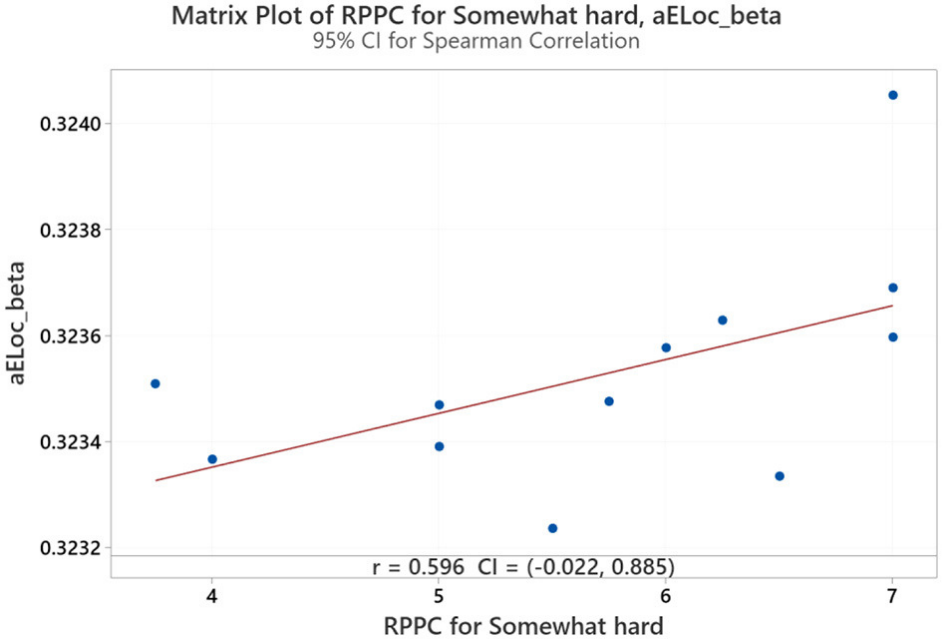
Correlation between the RPPC at somewhat hard applied force and local efficiency for beta coherence network (aELoc_beta).

**Figure 13:**
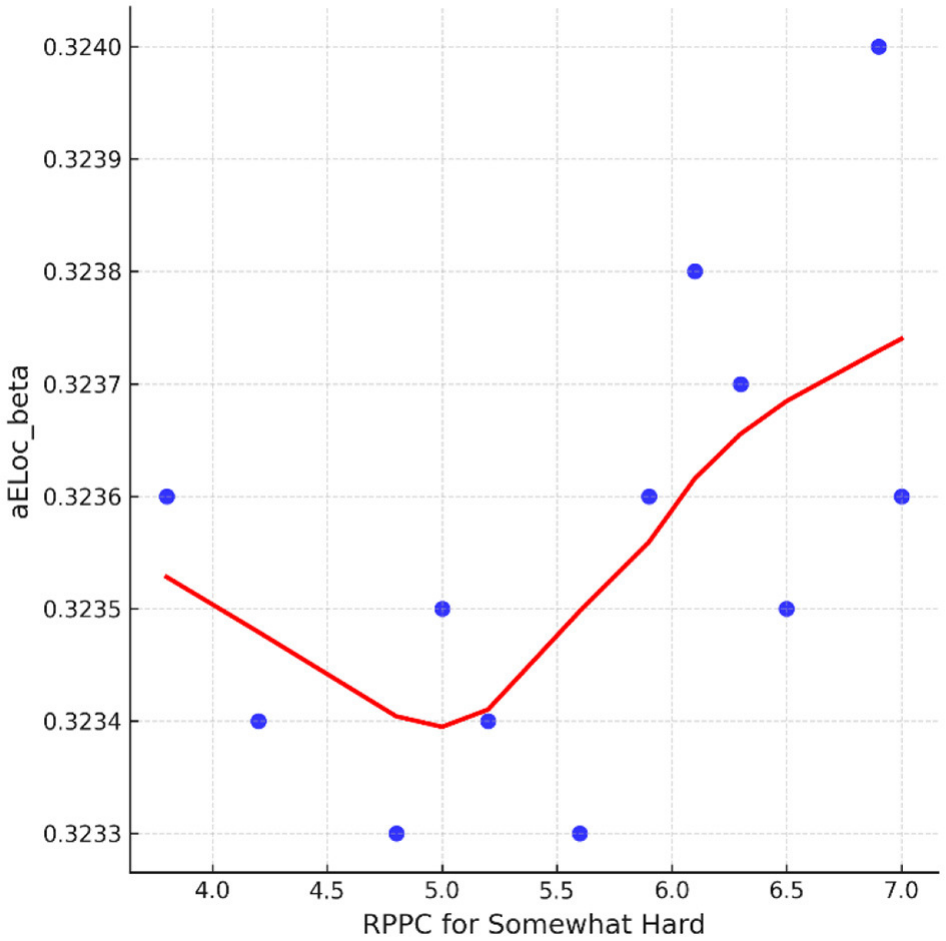
The LOESS curve of RPPC at somewhat hard applied force and local efficiency for beta coherence network (aELoc_beta).

On the contrary, at the somewhat hard applied force level, positive correlations were found between comfort scores and local efficiency for beta coherence (Figure 12). No significant correlations were found between network indices and the other force applied forces, including hard, light, and extremely light flexion levels. LOESS curve at Figure 13 was developed revealing a quite flat, indicating a minimal trend for non-linearity.

## 5 Discussion

The study aimed to explore the correlation between network indices and subjective rating of perceived comforts (RPPC) in response to physical activity. To achieve this, it was crucial to quantify the association between RPPC and the applied forces concerning different force levels. In addition to the association between the applied forces with network indices. Participants were required for an arm flexion task with different force levels to rate their physical comfort using a subjective comfort rating scale (Karwowski, 2018). Using the EEG technique, Neural data were collected and quantified using network indices. The study focused on quantifying the alpha and beta frequency bands since they are the most frequently examined in physical activity demandable tasks. For instance, the power for the alpha and beta bands increases after exercise (Mima et al., 2000), mainly in prefrontal and motor cortices (Maceri et al., 2019). Similar results were found after an intense exercise (Bailey et al., 2008). However, Robertson and Marino (2015) found an increase in the alpha wave at the prefrontal but maintained for motor cortices.

Different methods were used to quantify the brain function in physical activity, and these methods have been highly successful. For example, the coherence between EEG and electromyography has been widely used to investigate the synchronization between muscle activity and motor cortex during motor tasks (Cisotto et al., 2018). This method has shown successful results in rehabilitation studies for improving the quality of life (Kim et al., 2017; Nakano, 2021) and artifact detection (Luu et al., 2024).

Recent advances in neural signal analysis methods have made it possible to analyze the EEG data at a source level, especially after establishing LORETA‘s families (Pascual-Marqui, 2007). This led researchers to evaluate the functional brain connectivity of EEG data (Stam and Reijneveld, 2007) such as in arm exertion task (Ismail et al., 2022), cycling task (Tamburro et al., 2020; Lin et al., 2021), exercise-induced modulations (Büchel et al., 2021), combining working memory and exercise task (Porter et al., 2019), and motor imaginary task (Alanis-Espinosa and Gutiérrez, 2019). The novel contribution of our study was to explore the brain network indices associated with the human perception of physical comfort in static force flexion tasks. EEG coherence were previously obtained for alpha and beta networks using eLORETA (Ismail and Karwowski, 2023). Brain network indices were calculated using the Brain Connectivity Toolbox (http://www.brain-connectivity-toolbox.net) (Rubinov and Sporns, 2010), for the alpha and beta network at each applied force level. For this purpose, the correlation between the following was calculated and discussed. As some brain network indices do not relate to perception of physical comfort in a uniform manner, LOESS smoothing trends were applied to explore the possibility that the relationship between comfort and neurophysiological responses may not follow a strict linear pattern.

### 5.1 The correlation between RPPC and applied forces

Based on our explorative findings, we observed that as the difficulty of the force required for an arm flexion task increase, the RPPC decreases (i.e., comfort decreases). Similar to our findings, Lindegård et al. (2012) found a significant association between poor perceived comfort and the increase of upper extremity disorders. A LOESS curve fit revealed a nonlinear pattern, a LOESS curve was developed, indicating a nonlinear but mostly downward trend, reinforcing that comfort and force have an inverse relationship—but not strictly linear.

### 5.2 The correlation between the applied forces and network indices

A positive correlation between the extremely hard applied force and global efficiency for alpha network was observed. This finding aligns with studies that focus on the effect of physical task complexity on brain network indices. During an incremental-intensity exercise, the network efficiency first increases at low-moderate conditions and then decreases at exhaustive exercise conditions (Porter et al., 2019). The result might suggest that the brain is more efficient in transmitting information during a high demandable force task but not an exhaustive exercise conditions (Gu et al., 2023). A more efficient network is associated with enhanced cognitive processing (Stanley et al., 2015). Vigasina et al. (2023) found an increase in the global efficiency of the alpha band for the left hemisphere during a hand movement voluntary task, demonstrating that it might be connected to executive functions. Furthermore, authors support the existence of a small world network structure during hand movement voluntary task. Therefore, at the highest applied force levels, the increased cognitive and motor demands may lead to a shift in neural processing patterns, indicating a state of cognitive effort to sustain task performance despite low comfort. To further investigate the relationship between perceived task comfort and alpha coherence, we applied LOESS smoothing curve resulting in a nonlinear consistent upward trend.

A negative correlation was found between the light force and path length for the beta network. Generally, a reduction in the network path length suggests a faster information transmission between brain regions (Latora and Marchiori, 2003). Since beta frequency is more related to cognitive function (Von Stein et al., 1999), the reduction of the path length for the beta band reflects a faster cortical activation during an extremely hard force task. Our findings are similar to a recent article that showed an improved cognitive function performance during physical activity (Rodríguez-Serrano et al., 2024). Furthermore, a recent study by Yuk et al. (2024) observed an increase in frontal beta activity with higher resistance exercise, concluding that these potential changes are an indication of changes in mood-related symptoms. Therefore, a lower applied force a localized when comfort remains high the brain maintains an efficient but localized communication pattern.

### 5.3 A correlation between RPPC and network indices

The negative correlation between RPPC and global efficiency of alpha coherence at extremely hard force levels suggests that as comfort decreases, the brain's network becomes more efficient in global information transfer, possibly indicating high cognitive functional due to discomfort. A study concluded that increased global efficiency in the alpha network during a cycle exercise is attributed to increased alertness and readiness to respond to stimuli during exercise (Tamburro et al., 2020). Accordingly, perceiving a low-comfort physical task might indicate an increase in the alertness of the brain activity. Lastly, positive correlation between RPPC and local efficiency of beta coherence at somewhat hard force levels suggests that higher comfort is associated with more effective localized processing, reflecting a more balanced neural response during s doomist hard exertion level. A mental task study that used network indices to differentiate between a mental task and a resting task indicated a high local efficiency in beta frequency during a play task compared to a resting task (Huang et al., 2016). The authors justify that high local efficiency during the play task results from the involvement of the task execution; thus, the brain network shows a prominent level of internal organization. Consequently, we also may suggest a more concentrated attentiveness in perceiving somewhat hard physical comfort. The interpretations drawn from this study remain hypothetical and based on previous literature findings and should be confirmed with mediation analysis or causal modeling techniques.

## 6 Study limitations and future consideration

The application of network indices in engineering applications is gaining attention, but their relationship to behavior still needs to be better understood. Certain relationships remain unclear, such as whether the increase in global efficiency enhances cognitive functioning performance. Is the high local efficiency related to better cognitive functioning processing? Does the relationship differ with other connectivity measures, such as age, gender, task intensity, force difficulty level, mental or physical workload, etc.? A previous study highlights the importance of contextual exercise variables (Büchel et al., 2021). Despite the significance of previous research findings, a gap remains in the literature. The current study is crucial step to providing a pathway to these unclear behavioral relationships.

Our study is the first to compute the correlation between brain network indices and physical comfort during a physical task that requires different arm flexion force levels for female participants. The findings provide novel neurophysiological metrics for assessing ergonomic comfort perception. The study contributes to the current body of knowledge by providing empirical evidence that perception of physical comfort is associated with brain network indices. This attempt could provide benefit to understanding physical comfort perception not just based on subjective scales but also through real-time neural monitoring. Real-time monitoring provides benefits for break rescheduling and real time adjustment to workload level. The findings of the study would also enhance ergonomics and workplace design by minimizing the excessive flexion force, particularly in repetitive or high-force demandable tasks to ensure comfort threshold during physical task execution and prevent WMSDs. Consequently, the approach will help in minimizing employees with high risk to overexertion injuries, muscle fatigue and strain.

This work underscores the need for further research, particularly increasing the sample sizes and considering other experiments with male participants (Yuk et al., 2024). Although previous EEG studies have included modest number of participants (Gutmann et al., 2015; Churchill et al., 2016; Zhang et al., 2021; Wu et al., 2025) increasing the sample size and more diverse participants is better for further generalization across populations. This study is among the first to investigate the relationship between brain responses and comfort perception during exertion tasks, and our results are preliminary. Significant differences in brain results were found in previous studies related to gender difference (Corsi-Cabrera et al., 1993; Cantillo-Negrete et al., 2016; Hashemi et al., 2016; Cave and Barry, 2021). Additionally, significant differences in comfort perception, biomechanical differences, and judgments between gender were observed (Karwowski, 1991; Rahman et al., 2023). We also stress the importance of considering age differences in future studies, as age is associated with different cognitive shifts (Stanley et al., 2015). Although the current study was conducted in a temperature-controlled laboratory to ensure data quality and consistency due to brain signal artifacts, we recognize that this might impact the ecological validity of the findings. Future research should expand to more realistic or occupational settings. This expansion will provide a deeper understanding of how environmental variables influence neurophysiological responses and comfort perception during physical tasks. Additionally, some psychological factors (e.g., stress, fatigue, motivation) were not accounted for by the study, however, they may influence comfort ratings or EEG results thus future work may consider mood assessments scales.

Many previous studies have assessed brain functional connectivity in cognitive complexity tasks (O'Connell and Basak, 2018), with minimal focus on physical activities related to work, not just sports and exercise research. The literature offers a variety of methods to quantify rhythmic functional connectivity (Bastos and Schoffelen, 2016), and we urge the consideration of these different methods to further our understanding of brain network indices. While the current study primarily focuses on the alpha and beta frequency bands, which are associated with motor activity and sensorimotor processing. Future work should consider theta and gamma bands as they are critically involved in high cognitive processing, attention studies, working memory, and mental fatigue. The nonlinear patterns observed from the LOESS curve highlight the complexity of brain-comfort relationships indicating that brain responses are not always linear to physical demand. The findings are critical neuroergonomics research demonstrating that future studies should consider larger and heterogeneous samples using advanced statistical approaches such as mixed-effects models or mediation analysis to better capture brain's response. Future studies should employ more comprehensive statistical models such as partial correlations and multiple regression analysis accounting into account the effect of confounders such as body proportions (e.g., limb length), and muscle mass distribution.

Finally, results from this study might be integrated with novel mechanical assist devices such as exoskeletons for reducing excessive exertion levels to maintain comfort, especially in applications that requires prolonged force application. Moreover, future studies might combine artificial intelligence technology for creating an intelligent and adaptive workstation setup based on real-time neuro physiological feedback.

## 7 Conclusions

The present study investigated the correlation between (a) the rate of perceived physical comfort [RPPC and isometrically exerted forces (IEF)] in an arm flexion task, (b) IEF and network theory indices, and (c) RPPC and network theory indices. Results for (a) showed a significant negative correlation between RPPC and the applied forces. Results for (b) showed a positive correlation between the IEF and global efficiency for the alpha network coherence during an extremely hard arm flexion task. However, during a light force flexion task, a negative correlation with the path length of the EEG-beta coherence network was found. The above indicates that highly demanding arm flexion tasks result in a more efficient brain network activity, especially in transmitting information related to cognitive processing. Results for (c) showed a negative correlation between RPPC and global efficiency for alpha coherence during an extremely hard task. On the other hand, a positive correlation was found between RPPC and the local efficiency of the beta coherence network during a somewhat hard flexion task. Accordingly, we conclude that the brain network demonstrates high level of internal organization for arm flexion tasks perceived as highly comfortable. The current findings provide important insights into understanding the neurophysiological mechanism of human perception of comfort in physical activities.

## Data Availability

The datasets presented in this article are not readily available because the participants of this study did not give written consent for their data to be shared publicly, so due to the sensitive nature of the research supporting data is not available. Requests to access the datasets should be directed to wkar@ucf.edu.
